# Draft Genome Sequence of the *Streptococcus pneumoniae* Avery Strain A66

**DOI:** 10.1128/genomeA.00697-15

**Published:** 2015-06-25

**Authors:** Christoph Hahn, Ewan M. Harrison, Julian Parkhill, Mark A. Holmes, Gavin K. Paterson

**Affiliations:** a School of Biological, Biomedical and Environmental Sciences, University of Hull, Hull, United Kingdom; b Department of Veterinary Medicine, University of Cambridge, Cambridge, United Kingdom; c The Wellcome Trust Sanger Institute, Hinxton, United Kingdom

## Abstract

We have used HiSeq 2000 technology to generate a draft genome sequence of *Streptococcus pneumoniae* strain A66. This is a common study strain used in investigations of pneumococcal bacterium-host interactions and was used in the seminal genetic studies of Avery et al.

## GENOME ANNOUNCEMENT

*Streptococcus pneumoniae* is a prominent human pathogen throughout the world, particularly as a cause of pneumonia, meningitis, bacteremia, and otitis media. *S. pneumoniae* strain A66 (NCTC 7978) and derivatives, such as A66.1, are virulent in mice and have been used by many laboratories for *in vivo* studies examining host immune responses (1, 2), preclinical vaccination evaluation (3–5), the development of novel therapies (6, 7), studies on virulence mechanisms (8, 9), and the use of a bioluminescent form to track infection *in vivo* (10, 11). It has also been used in a variety of *in vitro* studies, notably including the work of Avery et al. (12). To facilitate the use of A66 as a study strain, we present here its draft genome sequence. DNA was harvested from a minimally passaged culture from the National Collection of Type Cultures, where the strain was deposited in 1949 from the Rockefeller Institute for Medical Research, and sequenced using Illumina HiSeq 2000 technology.

Assembly was performed using Velvet software (13), and the assembled contigs were reordered and oriented by alignment using Mauve software (14) with *S. pneumoniae* OXC141 as a reference. The assembly consisted of 159 contigs, with an *N*_50_ of 53,248 bp. It was automatically annotated using RAST (15) and compared to strains TIGR4 and OXC141 using the Artemis Comparison Tool. The A66 draft genome was 1,983,415 bp in length with a GC content of 39.7%. Annotation found 2,087 coding sequences, including the genes for pneumolysin, the serotype 3 capsular locus, neuraminidase A, choline binding protein A, hyluronidase, ZmpA, and zinc metalloprotease B. However, A66 lacked the *rlrA* pilus locus, *psrP* locus, and *zmpC*. The genome-derived multilocus sequence type is ST387 (16). No acquired antimicrobial resistance genes were identified in the genome sequence using ResFinder version 2.1 (17), and the strain was phenotypically susceptible to all antibiotics tested using Vitek 2 (card AST-ST01).

The availability of a draft genome sequence for A66 will greatly facilitate its value as a model strain for investigating pneumococcal biology.

### Nucleotide sequence accession numbers.

This whole-genome shotgun project has been deposited in DDBJ/ENA/GenBank under the accession number LN847353. The version described in this paper is the first version, LN847353.1.
